# Wnt signaling pathways in urological cancers: past decades and still growing

**DOI:** 10.1186/1476-4598-11-7

**Published:** 2012-02-10

**Authors:** Shahana Majid, Sharanjot Saini, Rajvir Dahiya

**Affiliations:** 1 Department of Urology, Veterans Affairs Medical Center, San Francisco and University of California San Francisco, 4150 Clement Street, San Francisco CA 94121, USA; 2 Professor and Director, Urology Research Center (112 F), Veterans Affairs Medical Center and University of California at San Francisco, 4150 Clement Street, San Francisco CA 94121, USA

**Keywords:** Wnt pathway, Kidney, Prostate, Baldder Cancer, MicroRNAs

## Abstract

The Wnt signaling pathway is involved in a wide range of embryonic patterning events and maintenance of homeostasis in adult tissues. The pathological role of the Wnt pathway has emerged from studies showing a high frequency of specific human cancers associated with mutations that constitutively activate the transcriptional response of these pathways. Constitutive activation of the Wnt signaling pathway is a common feature of solid tumors and contributes to tumor development, progression and metastasis in various cancers. In this review, the Wnt pathway will be covered from the perspective of urological cancers with emphasis placed on the recent published literature. Regulation of the Wnt signaling pathway by microRNAs (miRNA), small RNA sequences that modify gene expression profiles will also be discussed. An improved understanding of the basic genetics and biology of Wnt signaling pathway will provide insights into the development of novel chemopreventive and therapeutic strategies for urological cancers.

## Background

During embryogenesis, cells often acquire new identities as they migrate to new locations. Many of these morphogenetic changes are induced by extracellular ligands and their receptors [1-3]. Signaling events outside the cell act as positive or negative regulators of signaling pathways. This is particularly true for proteins with key functions in development, such as bone morphogenetic protein (BMPs) Hedgehog and Wnt. Various factors can interact with these proteins outside the cell, modulating their activity or altering their structure [4-10]. Wnt proteins, which are found in animals from hydra to insects, worms and vertebrates, are involved in a wide range of embryonic patterning events and maintenance of homeostasis in adult tissues [8,9,11-13]. One of the most striking effects of Wnt proteins is their ability to induce formation of a new embryonic axis in metazoans ranging from *Hydra *to *Xenopus *[14,15]. Defects in this pathway have been shown to cause various embryonic abnormalities in *Drosophila *and animal models and have been implicated in human cancers. Other signaling pathways important in embryonic pattern formation include the Nothch pathway and the tyrosine kinase receptor/Ras pathways [16] and those headed by members of the transforming growth factor (TGF)-β superfamily [17,18]. Instances of crosstalk between the embryonic signaling pathways notch, wnt, or Hh and other signaling pathways have been reported in a variety of cell types [19-21]. Although aberrant activation of an individual pathway may result in tissue specific carcinogenesis, these pathways rarely operate in isolation. Crosstalk between signaling pathways has the potential to profoundly add to the complexity of cellular responses to external stimuli. Various reports indicate crosstalk between Wnt signaling and other key cancer pathways regulating apoptosis, angiogenesis, proliferation, migration, invasion and metastasis [12,22-25].

Wnt-1, the first member of Wnt family protein was initially identified independently as a *Drosophila *segment polarity gene Wingless (Wg) and the murine protooncogene Int-1 [26]. The term Wnt was derived from a combination of Wingless and Int-1. Since the discovery of Wnt-1, multiple Wnt members have been found throughout the animal kingdom and the human genome encodes 19 Wnt genes [9]. For a wealth of information on Wnt signaling in general and a comprehensive list of Wnt target genes in particular, we direct the readers to the Wnt Home Page posted by the Nusse lab (http://www.stanford.edu/~rnusse/wntwindow.html). Intensive studies from past decades have identified essential components of signaling pathways by which Wnt proteins relay their signals into intracellular responses [9,27]. Wnt proteins can transduce their signaling through distinct intracellular routes which can be divided into two pathways as either 'canonical or 'non-canonical" Wnt pathways. The best understood canonical pathway utilizes nuclear β-catenin as an ultimate effector, leading to changes in gene expression that regulates cell proliferation, differentiation and survival, etc. In contrast, non-canonical pathways signal via a β-catenin -independent mechanism, generally resulting in changes in cell polarity and movement [28-30].

Early evidence of involvement of the Wnt pathway in cancer came from isolation of *Wnt-1 *as *Int-1*, a gene activated by nearby integration of the mouse mammary tumor virus in a mammary tumor model [31]. Oncogenic potential was also assessed in cultured mammalian cells, such as C57MG and CH310T1/2, where expression of the proto-oncogenic Wnts resulted in morphological transformation [32,33]. These cells are transformed by Wnt-1, Wnt-2, Wnt3a but not by Wnt-4, Wnt-5a, and Wnt-6. The transforming Wnt genes also promote the accumulation of β-catenin in some cultured mammalian cells [34]. Many mutations that promote constitutive activation of the Wnt signaling pathway lead to cancer. Individuals with Axin2 mutations display a predisposition to colon cancer [35]. Moreover, the best-known example of a disease involving a Wnt pathway mutation that produces tumors is familial adenomatous polyposis (FAP), an autosomal, dominantly inherited disease in which patients display hundreds or thousands of polyps in the colon and rectum. This disease is caused most frequently by truncations in APC, which promote aberrant activation of the Wnt pathway leading to adenomatous lesions owing to increased cell proliferation [36,37]. Mutational loss of APC function activates the Wnt transcriptional response by stabilizing β-catenin. Most sporadic colorectal tumors also involve constitutive activation of the Tcf-mediated Wnt transcriptional response, due to either loss of *APC *or stabilizing oncogenic mutations in β-catenin [13,38]. Loss-of-function mutations in Axin have been found in hepatocellular carcinomas [39]. These examples demonstrate that the uncoupling of normal β-catenin regulation from Wnt signaling control is an important event in the genesis of cancers. In renal cancer Wnt signaling has been found to contribute to disease development by influencing apoptosis [40]. These properties of the Wnt pathway were found to be mediated in part by splicing isoforms of TCF, since the lack of exon 15 in one human TCF is associated with reduced levels of expression of the anti-apoptotic factors Bcl2 and Bcl-XL and the pro-apoptotic factor Bak [40]. To further illustrate the role of Wnt signaling pathway in cancers, we will review this pathway below in the context of urological cancers.

### Wnt signaling in renal cancer

#### Canonical Wnt/β-catenin signaling

**Figure 1:**
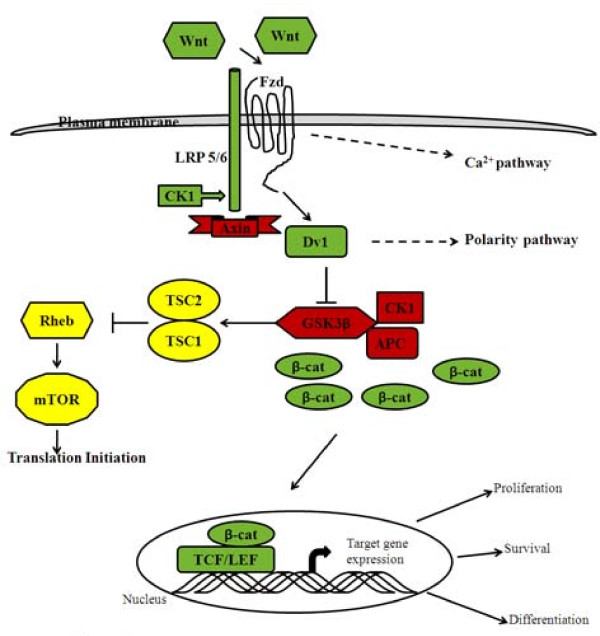
**Schematic representation of the Wnt signaling pathway in cancer cells**. In the presence of active Wnt, β-catenin accumulates in the cytoplasm, then localizes to the nucleus, and activates transcription together with TCF/LEF transcription factors. Negative regulators are depicted in red and positive regulators in green. Activation of the mTOR pathway is also directly regulated by Wnt-dependent downregulation of GSK3 kinase activity which is depicted in yellow.

Renal cell carcinoma (RCC) is the most common type of kidney cancer, accounting for 90% of all kidney cancers. It can be further classified into clear cell (ccRCC,80%), papillary (10-15%), chromophobe (5%), collecting duct (very rare) and a remaining unclassified group (5%) [41]. The ultimate effector of canonical Wnt signaling is the transcriptional coactivator β-catenin, which is emerging as a key molecule in the pathogenesis of renal cancer. Under normal conditions β-catenin levels in the cell cytoplasm are kept low as it is continuously degraded by the ubiquitin-proteasome pathway. β-catenin is marked for degradation by a multi protein degradation destruction complex that directly interacts with other components, like adenomatous polyposis coli (APC), glycogen synthase kinase3-B (GSK3-B) and casein kinase 1α (CK1-α) [9,42]. Prior to degradation, the NH_2_-terminus of cytosolic β-catenin is constitutively phosphorylated by a dual kinase mechanism. CK1-α phosphorylates β-catenin at Ser45, and this priming phosphorylation results in subsequent phosphorylaion by GSK3-B at residues Thr41, Ser37, and Ser33 [43,44]. β-catenin that is phosphorylated at residues 37 and 33 is ultimately recognized by the B-TrCP (B-transducing repeat containing protein), a component of a dedicated E3 ubquitin ligase complex [45]. Thus β-catenin gets ubiquitinated and subsequently undergoes rapid degradation by the 26S proteasome complex [46]. Wnt positively regulates β-catenin, inhibiting its phosphorylation, ubiquitination and degradation. Stabilized β-catenin enters the nucleus and together with a member of the LEF-TCF (lymphoid enhancer-binding factor 1-T cell specific transcription factor 7) family of transcription factors, activates target genes such as the *Myc *oncogene [47] (Figure 1). *Myc *also shows copy number amplification in a subset of primary ccRCC [48] and papillary RCC [49]. Though β-catenin -activating point mutations are rare in RCC [50], APC deficiency caused renal tumors in mice, presumably via the resulting elevated levels of β-catenin in mice [51]. Wnt is also thought to mediate its effect on cell growth and tumor promotion by activating the mTOR pathway [52]. TSC2 is sequentially phosphorylated by AMPK and GSK3 for its activation and subsequent inhibition of mTOR. Wnt activates the mTOR pathway by inhibiting GSK3 [52] (Figure 1). Altered expression of certain frizzled receptor (Fzd) family members and their downstream targets were found in renal carcinogenesis [53]. Increased expression of Fzd5 and Fzd8 both at mRNA and protein levels in renal carcinoma samples compared to normal samples. Kidney tumor tissue arrays showed Fzd5 membrane staining in 30% of clear cell carcinoma, with nuclear cyclin D1 showing a strong correlation with the Fzd5 membrane labeling. Wnt/β-catenin pathway activation was investigated by looking at the expression of various target genes, cMyc, cyclin D1 and peroxisome proliferator-activated receptor δ (PPAR δ), that have been reported to be upregulated in active pathway. The authors conclude that increased expression of Fzd receptors may have a role in active Wnt pathway in renal carcinogenesis [53]. Chakraborty et al. reported that Wnt receptors Frizzled (Fzd 1/2/4, 5, 7-10) and co-receptors low-density lipoprotein receptor-related proteins 5/6 (LRP5/6) were upregulated by chronic in vivo cadmium exposure in mice. Upregulation of Wnt signaling components induced by cadmium was corroborated by increased expression of Wnt target genes c-Myc and cyclin D1 that are involved in cell proliferation and survival, and the multidrug transporter P-glycoprotein Abcd1b, which promotes malignancy. Epithelial-mesenchymal-transitional markers Twist, fibronectin and collagen I were also upregulated suggesting that cadmium induced activation of the Wnt signaling pathway in renal epithelial tissues may lead to cancer [54].

The genetic basis of RCC is associated with the von Hippel-Lindau (VHL) tumor suppressor gene, identified in 1993 [55]. This gene is found to be mutated in most RCC cases and is highly related to clear cell renal carcinoma [56]. Furthermore aberrant VHL is found in 75% of patients with sporadic RCC [57]. The product of VHL (pVHL), in the presence of oxygen, recognizes and interacts with members of the hypoxia-inducible factor (HIF) α family. VHL polyubiquitinates HIFα subunits in normoxic conditions. Once HIF1α is hydroxylated and ubiquitinated, it gets destroyed by the proteasome [58]. HIF regulates the expression of genes that respond to hypoxia, such as glucose transporter (Glut)1, transforming growth factor (TGF) α, erythropoietin and the proangiogenic genes, vascular endothelial growth factor (VEGF), platelet-derived growth factor (PDGF) (reviewed in ref. 53) [59-63]. The expression of these genes creates an environment that favors cell proliferation and angiogenesis. The significance of the VHL-HIFα interaction has been confirmed by a study which showed that transfection of wild type VHL in renal carcinoma cell lines lacking expression of the VHL gene was sufficient to suppress tumor growth [64]. Similarly another study showed that HIF was the principal mediator of hypoxia-inducible gene deregulation in the VHL-/- renal cells and attenuation of HIF was sufficient to suppress the tumor forming capacity of these cells in nude mice [65]. Peruzzi et al. discovered that β-catenin is degraded by the E3-ubiquitin ligase activity of VHL and loss of VHL enables HGF-driven oncogenic β-catenin signaling as a novel target for VHL, thus implicating Wnt signaling in the pathogenesis of renal cancer [66]. Linehan et al. suggested that loss of VHL could lead to combined de-repression of HIFs and β-catenin, which in turn might contribute to malignancy in ccRCC [67]. In a recent study it was shown that like HIF1α, HIF-2α also interacts with β-catenin but at a different site. HIF-2α was found to assemble with β-catenin/TCF and facilitate gene transcription. HIF-2α was found to be required for β-catenin activation in RCC cells and for their proliferation. The interaction between HIF-2α and β-catenin contributes to the unrestrained growth of tumor cells containing coactivated HIF-2α and β-catenin. The authors further show that the interaction of HIF-2 α and β-catenin oppose those of HIF1-α on β-catenin and cell growth, which suggests that the ratio of HIF-1α/HIF2α may determine cell growth when hypoxia and Wnt stimulation coexist [68]. Another VHL-interacting protein Jade-1 (gene for apoptosis and differentiation in epithelia) has been shown to be a novel E3 ubiquitin ligase that ubiquitinates β-catenin leading to its degradation. Jade-1 is positively regulated by VHL and is thought to function as a renal tumor suppressor [69,70]. Loss of VHL results in reduced levels of Jade-1 and increased levels of β-catenin, providing yet another mechanism by which VHL loss promotes renal tumorigenesis. The link between the Wnt pathway and kidney cancer can be established from the observation that the hypoxia-inducible protein-2 (HIG2) binds to the Wnt receptor Fz10 at its extracellular domain and induces transcription of Wnt signaling target genes. The HIG2 functions as a cell proliferation inducer and has been identified as a marker of RCC. HIG2 is also a target of the β-catenin/Tcf4 complex [71]. Further evidence for the activation of Wnt signaling pathway in RCC comes from the article of Kojima et al. which describes the homozygous deletion of *CXXC4*, a gene coding for Idax (an inhibitor of the Wnt signaling pathway) in aggressive RCC [72]. Wnt has a dual role in pathogenesis of RCC. It not only induces transcription through activation of β-catenin, but also stimulates translation and cell growth through activation of the mTOR pathway. In a recent study, Koji et al. reported that IGFBP4 activated the Wnt/β-catenin signaling pathway in RCC. Over-expression of IGFBP-4 promoted cell growth, invasion and motility in renal cancer cells along with the induction of MT-MMP and M-CAM which is a marker for tumor progression [73]. The tumor suppressor or promoter activity of the various components of the Wnt signaling pathway has been summarized in Table 1.

**Table 1:** Cancer suppressor or promoter activity of Wnt pathway components and microRNAs involved in urological cancers

Gene	Cancer suppressor activity	Cancer promoter activity
**Wnt components**		

β-catenin	-	36,68, 71, 123, 140

GSK3-β	12, 36	-

Frizzled receptors	-	53, 54, 71

sFRP1	86, 89, 90, 91, 103, 106, 137, 138	110

sFRP2	138	111

sFRP3	-	112

sFRP4	138	-

sFRP5	107, 138	-

WIF1	108, 118, 138, 139, 140	-

DKK1	23	129, 130

DKK2	109	-

DKK3	22, 138	-

DKK4	-	24

Wnt3a	-	144

Wnt5a	-	116, 119

Wnt7b	-	136

Wnt11	-	117

**MicroRNAs**		

miR-15a	144	-

miR-16a	144	-

miR-200 family	145	-

Numbers indicate references.

#### Non-canonical Wnt signaling pathways

The Wnt/Ca^2+ ^and Wnt/polarity, also known as Planar Cell Polarity (PCP) pathways are known as the "non-canonical pathways". Other non-canonical pathways include Wnt/Jnk and Wnt/Rho signaling [28,30]. The intracellular signal transduction cascades that have been identified in either canonical or non-canonical pathways are very different from each other, but the common initial step is the binding of a Wnt ligand to the cognate Fzd receptor. Depending on the pathway which is activated, the initiation signal will be transduced differently. This decision most likely depends on which Wnt ligand and Fzd receptor are present, as well as the cellular context [74]. The Wnt/Ca^2+ ^pathway regulates cell adhesion and motility, and is mediated through release of intracellular Ca^2+ ^upon Wnt stimulation [28,75]. Wnt-5a was the first identified Wnt ligand identified to signal down this pathway and was shown to require coupling to G-proteins [76]. Interestingly, the Wnt/Ca^2+ ^pathway activated by Wnt-5a, antagonizes the Wnt/β-catenin pathway [77-80]. In the Wnt/polarity pathway, Fzd functions to establish asymmetric cell polarities and coordinate cell shape changes and cellular movement [28,30,74]. Fzd regulates the activity of the small GTPases Rho and Rac through different domains of Dv1. Rho and Rac, in turn, regulate the activity of Rock and Jun N-terminal Kinase (JNK) respectively [81]. Aberrant activation of the Wnt/PCP signaling pathway in human cancer leads to malignant phenotypes such as abnormal tissue polarity, invasion and metastasis [82].

#### Wnt Antagonist and their epigenetic modulation

The Wnt signaling pathway antagonists have been studied extensively in development and their involvement in oncogenesis has been demonstrated. There are four families of Wnt antagonists that can be divided into two sub-groups according to their mode of action. The first group includes the secreted-Frizzled related protein (sFRP) family, Wnt-inhibitory factor (WIF-1), and Cerberus. They inhibit Wnt signaling by direct binding to Wnt molecules. The second group, consisting of the Dickkopf (DKK) family, inhibits Wnt signaling by binding to the LRP5/LRP6 component of the Wnt receptor complex [83-85]. In mammalian cells, sFRP1 has been found to specifically bind to Wnt-1 protein, but not Wnt-5a protein, and it modulated the Wnt-1 signaling. sFRP-1 efficiently inhibited the Wnt-1 mediated increase in cytoplasmic β-catenin levels as well as the Wnt-1 induction of transcription from a Lef/Tcf reporter gene [86]. However binding specificity may not relate to functional specificity, as SFRP-3 associated with Wnt-5a but was unable to inhibit its activity [87]. Even the significance of specific functional interactions might be suspect based on titration experiments with purified soluble sFRP-1. At low concentrations sFRP-1 enhanced signaling activity by soluble wingless protein, whereas at higher concentrations it was inhibitory [88]. The authors proposed high and low states of binding affinity that involved the carboxy-terminal heparin binding domain and the amino-terminal cysteine-rich domain of sFRP-1, respectively. Binding to the cysteine-rich domain might confer inhibition while binding to the carboxy-terminal region could facilitate presentation of active ligand to receptor. Thus sFRP-1 exerts a biphasic effect on Wingless (Wg) activity [88]. Reports from other investigators and recent publications from our laboratory show the biphasic role of sFRPs in renal cancer. Gumz et al. showed that stable re-expression of sFRP-1 in clear cell RCC cells resulted in decreased expression of Wnt target genes, decreased growth in cell culture, inhibition of anchorage-independent growth, and decreased tumor growth in athymic nude mice. Thus sFRP-1 acts as a tumor suppressor and its restoration attenuated the clear cell renal cancer tumor phenotype [89]. Other studies reported that sFRP-1 expression loss is a common event in renal cancer [90,91].

Abnormal promoter methylation of tumor suppressor genes contributes to tightly heritable gene silencing and can cause loss of gene function, which thereby contributes to tumorigenesis. Various Wnt pathway components that regulate proper WNT/β-catenin signaling are frequently disrupted in human cancers through either genetic or epigenetic alterations [92,93]. Constitutive activation of WNT/β-catenin signaling as a result of mutations in APC and β-catenin was first documented in both inherited familial adenomatous polyposis (FAP) [37,94] and sporadic colon cancers [38,95]. Mutations of pathway components including *APC, AXIN1/2 *and β-catenin are well established in colorectal [35,38,96,97], gastric [98], hepatocellular [39] as well as other tumors [99]. Most of the human cancers show elevated levels of nuclear β-catenin, a hallmark of active WNT/β-catenin signaling, although mutations of *APC, AXIN *or β-catenin are substantially less frequent. In renal cell carcinoma APC and β-catenin mutations are uncommon events [100,101]. Whereas, CpG island hypermethylation at the promoter of a gene is a common and early event in kidney tumorigenesis [90,102-105]. Independent studies have reported hypermethylation of atleast one of a set of different genes (VHL, p16/INK4a, p14ARF, APC, RASSF-1A, TIMp-3, MGMT, GSTP1, CDH1, and ARF RARbeta2) in over 95% of tumor samples representing all major biological and histological types, grades and stages compared to no methylation in corresponding normal renal or urethral tissues. Results have been found to correlate DNA sediment from pre-operative urine samples, serum and tissue [102-104] highlighting their biomarker potential. Functional loss of negative WNT regulators by epigenetic gene silencing [92] has been frequently reported to contribute to the activation or amplification of aberrant WNT/β-catenin signaling in tumors. CpG promoter hypermethylation has been often found in antagonists of the Wnt pathway, the SFRP family, WNT inhibitory factor-1 and DICKKOPF family members after comparing primary renal cancer samples to the corresponding normal renal tissues. The methylation levels of six Wnt antagonist genes (sFRP-1, sFRP-2, sFRP-4, sFRP-5, Wif-1, and Dkk-3) were significantly higher in renal cancer compared to normal renal tissues. sFRP-1 methylation was found to be a significant independent predictor of RCC. In RCC patients, the methylation results were identical in samples of tumor and serum DNA. In addition, the methylation status of Wnt antagonist genes in serum DNA was significantly correlated with tumor grade and stage showing their potential as useful epigenetic biomarkers [106]. Loss of sFRP-1 due to hypermethylation is common in renal carcinoma [103] than other cancers [90]. The expression of sFRP-1 was decreased 89% at the mRNA level and 75% at the protein level while the promoter was found to be methylated in 68% of RCC samples [90]. sFRP-5 was epigenetically suppressed in RCC and its overexpression induced apoptosis in renal cancer cell lines [107]. The Wnt inhibitory factor-1 (WIF-1) promoter was found to be hypermethylated in RCC and its over-expression inhibited Wnt activity and induces apoptosis in renal cancer cells [108]. The Dickkopf class of Wnt antagonists including DKK1, DKK2 and DKK3 are also epigenetically silenced in renal cancer and their over-expression induced apoptosis and inhibited renal cell growth *in-vitro *and *in-vivo *[22,23,109], whereas DKK4 was found higher in renal cancer compared to normal tissue samples and it activated the non-canonical Wnt pathway in renal cancer thereby promoting the invasive and migratory capability of renal cancer cells [24]. The biphasic effects of some Wnt antagonists and their potential to activate Wnt signaling have been demonstrated in some recent reports from our lab. Saini et al. reported that sFRP1 is related to invasiveness and metastatic behavior in RCC [110]. The authors showed that the invasive capability of a metastatic renal cancer cell line was decreased by attenuating sFRP1 with a concomitant decrease in the levels of metastasis related gene *MMP10 *[110]. sFRP-2 activated the Wnt pathway and promoted renal cancer growth [111] whereas sFRP-3 expression induced the *MMP-3 *and *ANGPT1 *genes in renal cancer and thus contributed to the invasive capability of RCC [112]. Uren et al. observed that sFRP-1 exerted a biphasic effect on Wnt activity increasing armadillo level at low concentrations but reducing it at higher concentrations. Depending on the expression levels and molecular, cellular and tissue context, the SFRPs may promote Wnt signaling by protecting Wnts from degradation or by facilitating Wnt secretion or transport [88]. Rubin et al. have reviewed some possible mechanisms to the contradictory behavior of Wnt antagonists [113]. The functional consequences of over-expression patterns of Wnt antagonists with regard to tomorigenesis are largely unknown and much work will be required to define the specific relationships that govern the interactions of the Wnts, sFRPs, Dickkopfs and Fdzs.

### Wnt signaling in prostate cancer

There is mounting evidence that aberrant activation of the Wnt pathway is frequently associated with tumor development, progression and metastasis in prostate cancer [114,115]. This review will mainly focus on the work that has been done after publication of these previous reviews. Increased levels of various Wnts and other molecules involved in regulation of Wnt signaling have been detected in prostate cancer. A recent study showed that Wnt-5a promotes the aggressiveness of prostate cancer [116]. The positive detection of Wnt-5a was correlated with high Gleason scores and biochemical relapse. Knockdown and over-expression of Wnt-5a reduced and stimulated, respectively, the invasion and migration activities of prostate cancer cells. Wnt-5a activated Jun-N-terminal kinase through protein kinase D (PKD) and the inhibition of PKD suppressed Wnt-5a-dependent cell migration and invasion. Wnt-5a induced the expression of metalloproteinase-1 through the recruitment of JunD and thus contributed to the more aggressiveness of prostate cancer [116]. These studies suggest that in prostate cancer, Wnt-5a may be a useful target for small molecule inhibitors. Deliberate, well-designed screening campaigns will be integral to this effort, using either in vitro, cell-based, or in vivo models. As was undertaken to discover synthetic Wnt-3a inhibitors, high-throughput screens that utilize cell lines containing pathway-specific reporters will likely be the primary means of identifying such compounds. Wnt-11 protein levels were found to be elevated in human prostate tumors compared to benign prostatic hypertrophy specimens and it induced neuroendocrine-like differentiation in prostate cancer cells. Wnt-11 promoted prostate cancer cell invasion and migration and it was required for prostate cancer cell survival [117]. Yee et al. reported that Wnt inhibitor WIF1 gene is down-regulated in prostate cancer cell lines through promoter hypermethylation. Restoration of WIF1 expression resulted in decreased motility and invasiveness of prostate cancer cells [118]. In a recent study, Takahashi et al. showed that the non-canonical Wnt signal stimulates development of prostatic tumors with AR hyperfunction. In a prostate cancer model using transgenic mice, the onset of prostatic tumorigenesis as well as tumor growth was significantly potentiated by introduction of an AR point mutation (AR T877A) into the prostate and genetic screening of mice identified Wnt-5a as an activator [119]. Zhao et al., [120] reported over-expression of hypoxia inducible factor-1 (HIF-1 α) stimulates the invasion potency of human prostate carcinoma cells through EMT pathway and inhibition of Wnt signal activity through β-catenin shRNA caused a reversal of the EMT induced by HIF-1α [120]. Another study showed that Wnt/β-catenin signaling has an important role in the progression of mouse prostatic intraepithelial neoplasia (mPIN) to prostate adenocarcinoma. Prostates of mice expressing SV40-large T-antigen (LPB-Tag) and the Wnt/β-catenin pathway resulted in invasive prostate adenocarcinoma. Active Wnt/β-catenin signaling induced Foxa2, a forkhead transcription factor, that was associated with the invasive phenotype in primary prostate cancer [121].

A number of previous studies have reported that β-catenin is a biomarker in prostate cancer. Although β-catenin stability is regulated by a multi-component destruction complex, mutational alterations of β-catenin or other components of the destruction complexes are rare in prostate tumors. Therefore, β-catenin may be regulated by another protein in prostate cancer. Somatic deletion analysis in prostate cancers revealed a 1.4-Mb candidate tumor suppressor locus on 8p23.1, which includes the Sox7 gene [122]. Sox7 protein expression was down-regulated in 47% of prostate adenocarcinomas whereas mRNA was down-regulated in 60% of snap-frozen tumors. The silencing of this gene was due to promoter hypermethylation in prostate cancer. Sox7 suppressed β-catenin mediated transcription by depleting active β-catenin [122]. Another study showed that combinatorial oncogenic mutations of K-ras and β-catenin drive rapid progression of prostate tumorigenesis to invasive carcinoma, characterized by the synergistic elevation of androgen receptor, cyclooxygenase-2 and c-Myc [123]. Yardy et al. [124] studied mutations in genes encoding Wnt pathway in prostate cancer clinical samples and cell lines. Abnormal patterns of β-catenin expression were observed in 71% of specimens suggesting Wnt pathway dysregulation. One APC mutation, two β-catenin gene mutations and 7 DNA sequence variations in the Axin gene were detected. Four different Axin polymorphisms were also found in cell lines [124]. Although this study does not provide definite evidence that the observed sequence changes alter protein function, it does discuss the potential functional relevance of these variants in prostate cancer progression. More than 50% of human prostate cancers overexpress ERG (v-ets avaian erythroblastosis virus E26 oncogene related gene). Activation of AR has been shown to induce ERG through AR/TMPRSS2-ERG fusion. ERG induction and nuclear translocation resulted in the activation of the Wnt signaling pathway and promoted invasive capacity of prostate cancer cells [125].

#### Wnt signaling and prostate cancer metastasis to bone

**Figure 2:**
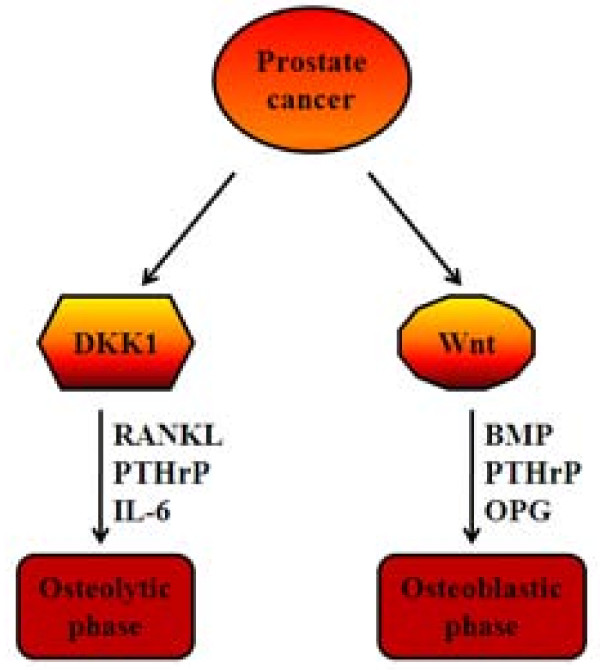
**Role of the Wnt signaling pathway in prostate cancer bone metastasis**. Prostate cancer cells have both osteolytic and osteoblastic potential. Early in skeletal metastasis, prostate cancer cells produce pro-osteolytic factors such as receptor activator of NFkB ligand (RANKL), interleukin-6 (IL-6) and parathyroid hormone-related protein (PTHrP) that stimulate osteoclastogenesis and also produce an inhibitor of osteoblastic activity, dickkopf-1 (DKK1). The resulting osteolytic activity releases growth factors from the bone and alters the bone microenvironment, which in turn alters the phenotype of prostate cancer cells. The prostate cancer cells start to produce osteoblastic factors such as bone morphogenetic proteins (BMP), PTHrP (which can act as an anabolic factor) and factors that inhibit osteclastogenic activity, such as, osteoprotegerin (OPG), which blocks RANKL. Additionally, DKK-1 expression is decreased resulting in the osteoblastic phase.

In addition to roles in the initiation and progression of the primary tumor, the Wnt pathway may also play key roles in the metastasis of prostate tumors, particularly to the bone [115,126,127]. Prostate cancer primarily metastasizes to bone, and the interaction of cancer cells with bone cells results in a local activation of bone formation (osteoblastic lesions). Bone morphogenetic proteins (BMP) and Wnts are mediators of prostate cancer induced osteoblastic activity (Figure 2). Wnt-3a and Wnt-5a administration or knockdown of DKK-1 induced BMP-4 and 6 expression and promoter activation in prostate cancer cells. Transfection of C4-2B cells with axin, an inhibitor of canonical Wnt signaling, blocked Wnt-3a but not Wnt-5a induction of BMP promoters. In contrast, Jnk inhibitor 1 blocked Wnt-5a but not Wnt-3a induction of the BMP promoters. Wnt-3a, Wnt-5a and conditioned medium from prostate cancer cells induced osteoblast differentiation *in-vitro *and pretreatment of prostate cancer cells with DKK1 diminished osteoblast differentiation. The authors concluded that prostate cancer promotes osteoblast differentiation through canonical and noncanonical Wnt signaling pathways that stimulate both BMP-dependent and BMP-independent osteoblast differentiation [128]. In the context of the bone micro-environment, Wnt antagonist DKK1 promotes the development of osteolytic lesions. Hall et al. [129] proposed that elevated DKK1 expression is an early event in prostate cancer and it may be involved in an initial osteolytic phase of prostate cancer metastasis to the bone. As prostate cancer progresses DKK1 expression declines, particularly in advanced bone metastases. This decline of DKK1 unmasks Wnt's osteoblastic activity and thus a shift to an osteoblastic phase occurs [129] (Figure 2). In a recent study, Thudi et al. [130] reported that DKK1 significantly increased Ace-1 subcutaneous tumor mass and the incidence of bone metastases after intracardiac injection of Ace-1 prostate cancer cells. The increase in tumor growth was associated with increased phopho46 c-Jun amino-terminal kinase by the Wnt noncanonical pathway. DKK1 decreased the Ace-1 osteoblastic phenotype of bone metastases via the Wnt canonical pathway evidenced by an inhibition of T-cell factor activity in murine osteoblast precursor ST2 cells [130]. Much work needs to be done to understand the role of Wnt signaling pathway activation and inhibition in both the metastatic prostate cancer cells and the osteoblasts in the surrounding lesions. Still, the Wnt pathway is now a promising target for the development of therapeutics that may interfere with prostate cancer metastasis to the bone.

### Wnt signaling in bladder cancer

While the role of Wnt signaling in bladder cancer has not been extensively studied, the studies done so far demonstrate that it plays a significant role in bladder oncogenesis. A recent study by Ahmad et al. showed that activation of Wnt signaling plays a critical role in driving baldder cancer and suggests that human bladder cancers which have high levels of Wnt and PI3 kinase signaling may be responsive to mTOR inhibition [131]. Another study from the same group reported that the Wnt signaling pathway is deregulated in approximately 25% of bladder cancer and a combined Ras mutation with an activating β-catenin mutation within the mouse bladder rapidly developed bladder cancers [132]. Evidence of activation of Wnt signaling in urothelial carcinoma also comes from other studies that identified frequent gene silencing of endogenous Wnt inhibitors [133] and less frequent APC mutations in bladder cancer [134,135]. Wnt-7b was found to be upregulated in papillary noninvasive carcinomas [136]. Stoehr et al. reported that in primary urothelial bladder tumors and cell lines, LOH at the APC gene locus was found in 10% of the informative cases. No mutations were found in either CTNNB1 or APC. All bladder cancer cell lines showed normal membranous β-catenin staining without evidence for nuclear or cytoplasmic accumulation. The authors concluded that alteration of APC and β-catenin are rare events in urothelial carcinomas [134]. The same group however found that 38% of bladder cancer samples showed loss of sFRP1 expression at the mRNA level whereas at the protein level loss or strong reduction of sFRP1 was observed in 66% of samples. The loss of sFRP1 was associated with higher tumor stage and grade and shorter overall survival. They reported that loss of sFRP1 was an independent indicator of poor survival in patients with papillary bladder cancer but not with muscle invasive bladder cancer [137]. Another study demonstrated higher promoter CpG hypermethylation and lower expression of mRNA transcripts for *sFRP-1, sFRP-2, sFRP-4*, and *sFRP-5, Dkk-3*, and *Wif-1 *genes in bladder tumor compared with normal bladder mucosa showing an inverse correlation. The methylation levels of *sFRP-2 *and *Dkk-3 *were found to be significant independent predictors of bladder tumors, whereas with *sFRP-1, sFRP-5*, and *Wif-1*, a trend towards significance was found as independent predictors [138]. Thus Wnt signaling appears to promote bladder cancer growth potentially in both noninvasive and invasive bladder cancer.

Gene silencing by DNA methylation downregulates expression of several secreted Wnt antagonists, including WIF-1 and DKK1. Wissmann et al. reported that 26% of bladder cancers showed reduction of Wnt inhibitory factor-1 (WIF-1) expression that correlated with higher tumor stage in bladder tumors [139]. Another group reported that the WIF-1 promoter was hypermethylated in bladder cancer that may contribute to the pathogenesis of bladder cancer through aberrant canonical Wnt/β-catenin signaling. Higher nuclear accumulation of β-catenin was observed that inversely correlated with the WIF-1 expression and the LOH close to the WIF-1 gene loci was found to be a rare event in bladder cancer [140].

### Wnt signaling and microRNA

MicroRNAs (miRNAs) are small, non-coding RNAs that have been found to regulate expression of 60% of all human genes through targeted repression of gene transcription and translation. They play important roles in a wide spectrum of biological processes, including development, proliferation, and apoptosis [141-143]. Investigations into the role of microRNA with regard to the Wnt pathway in urological cancers are in their infancy and only a few studies are available. MicroRNA-15a and miR-16-1 were reported to be tumor suppressors in prostate cancer. Both microRNAs form a cluster in chromosome region 13q14, which is frequently deleted in cancers. In advanced prostate cancer tumors, miR-15a and miR-16-1 levels were found to be significantly decreased with an increased expression of WNT-3a, Bcl2 and CCND1. Reconstitution of these microRNAs resulted in growth arrest, apoptosis and marked regression of prostate tumor xenografts through the down-regulation of WNT-3a, Bcl2 and CCND1 [144]. Another study demonstrated that the expression of miR-200 family members- miR-200a, miR-200b, and miR-200c was significantly downregulated in PC3 PDGF-D cells compared with PC3 Neo cells. Re-expression of the miR-200 family in prostate cancer cells led to the reversal of the epithelial-mesenchymal transtition (EMT) phenotype, which was associated with the down-regulation of ZEB1 and ZEB2 and concomitant increased expression of epithelial markers like E-cadherin, EpCAM and CRB3 [145].

Despite great advances in other cancers, miRNAs related to Wnt signaling remain largely uncharted with regard to urological cancers. Signaling complexes are highly dynamic, ephemeral and non-stoichiometric molecular ensembles and emerging evidence suggests that miRNAs translate into dose-dependent responsiveness of cells to signaling molecules such as Wnt, Notch etc. As such, signaling molecules are the ideal targets for the degree of quantitative fluctuations imposed by miRNAs. This might enable the multi-gene regulatory capacity of miRNAs to remodel the signaling landscape, facilitating or opposing the transmission of information to downstream effectors in an effective and timely manner. Much work needs to be done to decipher miRNA function with regard to Wnt signaling molecules in urological cancers.

### Wnt signaling inhibitors

Given the critical roles of Wnt pathway activation in the pathophysiology of many human diseases including cancer, interest in the development of Wnt signaling inhibitors has increased substantially. Several groups have identified various therapies and phytochemicals that either directly or indirectly disrupt β-catenin-mediated Wnt signaling. These agents include non-steroidal anti-inflammatory drugs, exisulind, vitamin A derivatives, endostatin etc. and phytochemicals such as flavanoids (genistein), curcumin, epigallocatechin-3-gallate (EGCG), resveratrol, lupeol, retinoids, lycopene and deguelin etc. (reviewed in ref. [74,146]). Different components of Wnt signaling pathway can be regarded as useful targets in preventing and treating cancer and the pharmacological modulation of Wnt target genes expression might require small molecules that can perturb multiple isoforms of a given Wnt pathway component. The identification of such small molecules is indeed a challenging endeavor. The first unbiased screen for Wnt pathway inhibitors was reported using a cell line stably transfected with a Wnt3a expression construct and TCF/LER-dependent firefly luciferase reporter [147]. Almost 200 000 compounds were surveyed and molecules that inhibit Wnt ligand production or responsiveness were discovered in this screen. The authors discovered two classes of small molecules that disrupt Wnt pathway responses; one class of molecules, benzothiazole-based inhibitors of Wnt production (IWPs), target the activity of Porcupine, a membrane-bound acyltransferase that is essential to the production of Wnt proteins, the other class is inhibitor of Wnt response (IWRs) that abrogates destruction of Axin proteins, which are suppressors of Wnt/beta-catenin pathway activity. Another group used a similar highthroughput chemical genetic screen to identify a small molecule, trifluoromethylphenylpyrimidine derivative called XAV939, which selectively inhibits b-catenin-mediated transcription [148]. XAV939 stimulates b-catenin degradation by stabilizing axin, the concentration-limiting component of the destruction complex. Using a quantitative chemical proteomic approach, authors discovered that XAV939 stabilizes axin by inhibiting the poly-ADP-ribosylating enzymes tankyrase 1 and tankyrase 2. Both tankyrase isoforms interact with a highly conserved domain of axin and stimulate its degradation through the ubiquitin-proteasome pathway [148]. These studies show the promise of small-molecules as next-generation chemotherapies. The clinical use of these agents is associated with certain risks and challenges. It is possible that chemical modulators of these developmental pathways will have unintended effects on tissue homeostasis and regeneration since these processes frequently recapitulate developmental mechanisms, for example, Wnt signaling is required for renewal of the intestinal epithelium. The effects of these inhibitors on normal adult physiology are likely to be reversible, and it is possible that dysregulated cells associated with human disease will be more sensitive to these compounds than healthy adult tissues. Technologies that help target these therapies to diseased cells could also help mitigate any adverse responses. However, extra caution should be exercised with treatments as they can lead to life-long developmental deficits. Despite these risks, small-molecule inhibitors of developmental signaling pathways provide new, long-awaited hope for many patients afflicted with diseases that currently lack effective treatments.

## Conclusion and perspectives

Given the similarities between embryonic growth control, dysregulated cell proliferation in development of cancer and the findings that Wnt signaling regulates kidney organogenesis, the Wnts were candidates for involvement in the development of kidney cancers and thus it has been widely studied in relation to kidney cancers so far. Intense efforts have also been made recently to examine the role of the Wnt pathway in prostate cancer. The Wnt signaling pathway and its key component β-catenin have also recently emerged as important players in bladder tumorigenesis. However, very few studies have been carried out to understand the biological role of this pathway in the pathogenesis of bladder cancer. The intent of this review was to highlight the studies performed around the Wnt signaling pathway in urological cancers and to promote its unexploited potential for drug design and biomarker use.

Recent data have shown that perturbations in Wnt signaling are involved in urological cancers. Tumor promotion by this pathway can proceed through a number of different genetic and epigenetic defects. In particular, a wealth of evidence implicates that chronic activation of β-catenin signaling is common in many tumor types. There is a clear need for new lead compounds targeting the Wnt/β-catenin pathway. These inhibitors may provide significant therapeutic benefit against a variety of human diseases in which the Wnt signaling pathway plays an important pathological role. Due to the emerging role of miRNAs in signal transduction, it also becomes apparent how the highly dose-sensitive nature of developmental signaling pathways like Wnt renders them prime candidates for miRNA regulation. Merging activity-based or expression-based screens with new RNA-based therapeutics may offer opportunities for targeting Wnt signaling pathways in cancer.

## Competing of interests

The authors declare that they have no competing interests.

## Authors' contributions

SM prepared the manuscript. SS assisted in the preparation and RD edited and modified this review article. All authors read and approved the final manuscript.

## Author's information

Dr. Shahana Majid serves as an Assistant Research Scientist in the Department of Urology at UCSF and the VA Medical Center. Dr. Majid's research interests include genetic and epigenetic studies and exploring the role of tumor suppressor genes in urological cancers. Currently, Dr. Majid's research explores the role of microRNA mediated gene activation and function in prostate, kidney and bladder cancers.

Dr. Saini is an Assistant Research Scientist in the Department of Urology at SFVAMC/UCSF. Her research is primarily focused on understanding the etiology of various urological malignancies, including kidney cancers. She is particularly keen on exploring the molecular basis of progression and metastasis of these malignancies. A molecular biologist by training, Dr. Saini has worked as a post-doc fellow at University of Massachusetts Medical School Worcester and SFVAMC/UCSF.

Dr. Rajvir Dahiya is a professor and Director of Urology Research Center at the UCSF/VAMC. Dr. Dahiya has a long-standing interest in molecular genetics and epigenetics of urological malignancies with a focus on developing novel biomarkers for the early diagnosis and prognosis of these cancers. Dr. Dahiya has published over 300 original research manuscripts in national and international journals and holds multiple oncology patents. Dr. Dahiya is a program director for many research projects.
